# Advances in the genetics of myasthenia gravis: insights from cutting-edge neuroscience research

**DOI:** 10.3389/fmed.2024.1508422

**Published:** 2025-01-07

**Authors:** Zheng Yixian, Wang Hai, Liu Xiuying, Yan Jichun

**Affiliations:** ^1^Zhangpu County Hospital, Zhangzhou, Fujian, China; ^2^Yue Bei People's Hospital, Shaoguan, China; ^3^The First People's Hospital of Qinzhou, Qinzhou, China; ^4^Ganzhou City People's Hospital, Ganzhou, Jiangxi, China

**Keywords:** myasthenia gravis, genetic susceptibility, genome-wide association study, transcriptome-wide association study, epigenetic mechanisms

## Abstract

Myasthenia gravis (MG) is an autoimmune disorder involving complex interactions between genetic and environmental factors. Genome-wide association studies (GWAS), transcriptome-wide association studies (TWAS), and other methods have identified multiple novel susceptibility loci and genes, providing crucial insights into the genetic etiology of MG. Moreover, the pivotal roles of epigenetic mechanisms, such as DNA methylation, histone modifications, and non-coding RNAs, in the pathogenesis of MG are gradually being unveiled. This review comprehensively summarizes the latest advances in MG genetic research, focusing on the discovery and validation of susceptibility genes, genetic heterogeneity and subtype-specific genetic factors, gene–environment interactions, epigenetic mechanisms, and progress in genetics-based diagnostic and prognostic biomarkers.

## 1 Introduction

Myasthenia gravis (MG) is an acquired autoimmune disorder characterized by skeletal muscle weakness and abnormal fatigability (1). MG is caused by autoantibodies targeting components of the postsynaptic muscle membrane at the neuromuscular junction. In most cases, antibodies against the acetylcholine receptor (AChR) can be detected, while other targets such as muscle-specific kinase (MuSK) and low-density lipoprotein receptor-related protein 4 (LRP4) have been discovered in recent years (2, 3). MG can be classified based on the location of affected muscles (e.g., ocular or generalized), age at symptom onset, and autoantibody profile. These criteria are crucial for optimizing the management and treatment of MG patients (4). In patients with anti-AChR antibodies, thymic abnormalities, immunoregulatory defects, and sex hormones are thought to play important roles. Genetic susceptibility may also influence disease occurrence (5, 6).

GWAS have identified multiple MG susceptibility loci, mainly involving immune-related genes such as human leukocyte antigen (HLA) and PTPN22 (7, 8). Additionally, epigenetic mechanisms, including DNA methylation, histone modifications, and non-coding RNAs, are gradually being revealed to play a role in the pathogenesis of MG (3).

## 2 Discovery and validation of MG susceptibility genes

In recent years, with the rapid development of molecular genetics and genomics technologies, the understanding of MG susceptibility genes has deepened. GWAS, TWAS, and other methods have been successively applied to MG genetic research, uncovering multiple novel susceptibility loci and genes, providing important clues for elucidating the genetic etiology of MG (9–12).

### 2.1 GWAS reveals novel MG susceptibility gene loci

A recent large-sample GWAS involving 1,873 AChR antibody-positive MG patients and 36,370 healthy controls identified 10 loci significantly associated with MG, including the previously reported PTPN22, TNFRSF11A, and HLA regions, as well as newly discovered loci such as 10p14 and 11q21 (9). Another GWAS meta-analysis covering 1,401 MG patients and 3,508 controls further confirmed the association of the TNFRSF11A gene and revealed the pathogenic role of the AGRN gene through gene functional enrichment analysis (12).

Additionally, some studies have attempted to explore the genetic heterogeneity of different MG subtypes. A retrospective cohort study conducted in North America, which included 1,032 AChR antibody-positive MG patients, found differences in genetic risk factors between early-onset MG (EOMG) and late-onset MG (LOMG) (11). A study by Korean researchers focused on ocular MG and GWAS results suggested that the PTPN22 and HLA-DQA1 loci were specifically associated with this subtype (13). These studies indicate that different clinical phenotypes of MG may have distinct genetic foundations.

While GWAS has successfully identified these genomic risk loci, understanding their functional implications requires additional approaches. Transcriptome-wide association studies (TWAS) complement GWAS findings by directly examining gene expression patterns, helping to bridge the gap between genetic variation and disease mechanisms (9, 12).

### 2.2 TWAS reveals the association of acetylcholine receptor subunit genes with MG

A TWAS study involving 1,873 MG patients utilized gene expression profiles from skeletal muscle, whole blood, and tibial nerve to identify expression quantitative trait loci (eQTLs) significantly associated with MG in the nicotinic cholinergic receptor α1 subunit (CHRNA1) and β1 subunit (CHRNB1) genes, respectively (14). Considering that AChR is the primary autoimmune target in MG, this result suggests that abnormal expression of genes encoding AChR subunits may be an important link in the pathogenesis of MG.

**Figure 1:**
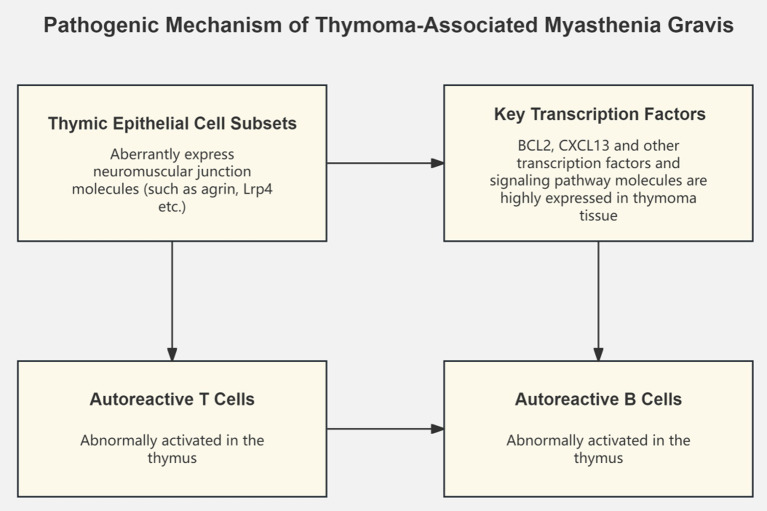
Pathogenic mechanism of thymoma-associated myasthenia gravis.

Concurrently, some studies have focused on transcriptomic changes in MG thymic tissue. By constructing a single-cell transcriptional atlas of MG-associated thymomas, researchers discovered the ectopic expression of neuromuscular junction molecules in a subset of medullary thymic epithelial cells, speculating that these abnormally expressing cell subsets may play a key role in the pathogenesis of MG by activating autoreactive T and B cells (7). Another study integrating thymoma tissue and normal thymus transcriptome data identified key transcription factors and signaling pathways such as BCL2 and CXCL13, which may be core mechanisms driving the development of MG thymomas (14). Recent single-cell transcriptomics studies have provided unprecedented insights into MG pathogenesis. Zhong et al. characterized the peripheral immune landscape in myasthenic crisis using single-cell RNA sequencing, revealing distinct immune cell states associated with disease severity (15). Additionally, Liu et al. identified novel therapeutic targets through single-cell analysis of immune cell subsets in pediatric MG (16), while Tian et al. demonstrated how B cell lineage reconstitution underlies CAR-T cell therapeutic efficacy in refractory MG patients (17) (Figure 1).

### 2.3 Functional annotation and bioinformatics analysis of MG-related genes

**Figure 2:**
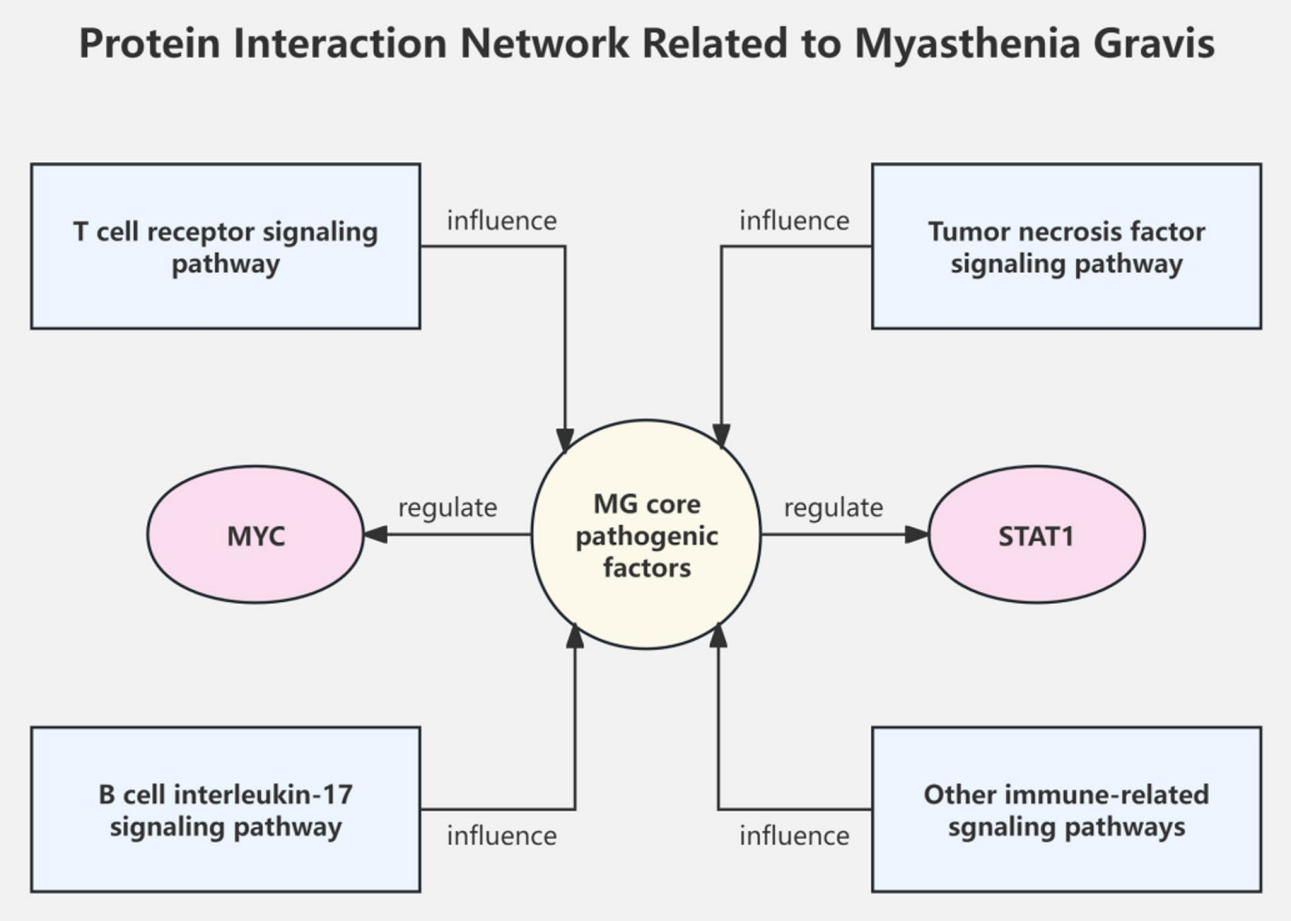
Protein interaction network related to myasthenia gravis.

Pathway enrichment analysis shows that risk genes discovered by MG GWAS are mainly involved in immune-related signaling pathways such as T cell receptor, tumor necrosis factor, and interleukin-17 (18–20). Protein–protein interaction network analysis reveals the important role of transcription factors such as MYC and STAT1 in regulating core MG pathogenic genes (20) (Figure 2).

Furthermore, a study integrating GWAS, TWAS, Mendelian randomization, and colocalization analysis identified 4 genes, including CTSH and CD226, and 3 proteins significantly associated with MG, validating their pathogenicity and suggesting that CTSH expression in Th2 cells is closely related to MG risk (14). A transcription factor-miRNA-gene feed-forward loop network constructed using bioinformatics algorithms predicts potential biomarkers and novel drug targets for MG from the perspective of epigenetic regulation (21).

Despite significant progress in the study of MG susceptibility genes, many challenges remain. The contribution of discovered genetic variants to the disease is limited and insufficient to fully elucidate the genetic risk of MG. The pathogenic roles of rare variants and structural variants warrant further investigation. There is an urgent need for large-sample, multi-center studies across different races and regions. Integrating multi-omics data to construct more precise genetic risk prediction models is an emerging trend.

## 3 Research on MG genetic heterogeneity and subtype-specific genetic factors

Different MG subtypes exhibit significant clinical heterogeneity. EOMG, LOMG, thymoma-associated MG, and anti-MuSK antibody-positive MG each have distinct characteristics, suggesting that their pathogenesis may involve different genetic mechanisms. In recent years, researchers have explored various MG subtypes from a genetic perspective, discovering some subtype-specific genetic variants and risk loci, providing important clues for unveiling the genetic basis of MG heterogeneity.

### 3.1 Differences in genetic risk factors between early-onset and late-onset myasthenia gravis

EOMG and LOMG exhibit significant differences in age of onset, clinical features, autoantibody profiles, and other aspects, suggesting that their genetic backgrounds may not be consistent. A next-generation sequencing study analyzed the human leukocyte antigen (HLA) genes of EOMG and LOMG patients in Italian, Norwegian, and Swedish populations, finding that the HLA-B*08:01 allele was the primary risk factor for EOMG patients, while the HLA-DRB1*15:01 allele was mainly associated with LOMG patients, indicating clear differences in immunogenetic susceptibility between the two subtypes (22). A study in the Spanish population found that HLA-DQB1*03:01 was a risk factor for EOMG, especially in female AChR antibody-positive patients with thymic hyperplasia (23).

In addition to the HLA region, the roles of some non-HLA gene loci also differ between EOMG and LOMG. PTPN22 and TNFAIP3 gene polymorphisms are mainly associated with EOMG, while ZFAT gene variants may be specific risk factors for LOMG (24). Moreover, epigenetic factors such as long non-coding RNAs and CircRNAs may also play different regulatory roles in the pathogenic mechanisms of the two subtypes (13). These studies demonstrate that EOMG and LOMG have distinct genetic susceptibilities, supporting their consideration as independent subtypes in MG genetic research. Further exploration of the differences in their pathogenic mechanisms will facilitate more precise subtype diagnosis and treatment.

Juvenile MG is an MG subtype with an early age of onset (usually <18 years) and mainly affects extraocular muscles. Some studies suggest that juvenile MG may have unique genetic susceptibility factors. A study involving 54 Chinese juvenile MG patients found that 17 (31.5%) carried TTN gene mutations, significantly higher than the control group, suggesting that TTN mutations may be potential therapeutic targets for juvenile MG (25). A cohort study in Turkey found that adolescent females were more prone to developing generalized juvenile MG with a more severe clinical course, requiring more aggressive treatment strategies, including thymectomy, which may be related to factors such as changes in hormone levels during puberty (26).

### 3.2 Specific genetic alterations in thymoma-associated MG

Thymoma is the most common tumor complication of MG, with approximately 10–20% of MG patients having concurrent thymoma, and 30–40% of thymoma patients developing MG symptoms, suggesting a close pathogenic link between the two (27). The latest research has discovered that the thymic tissue of thymoma-associated MG (TAMG) patients contains a unique subset of medullary thymic epithelial cells that ectopically express neuromuscular junction molecules such as agrin and Lrp4, which may be involved in the development of MG by activating autoreactive T and B cells (28). Additionally, dysregulated expression of apoptosis-related genes such as p53 and Bcl-2, and overexpression of chemokines such as CXCL13 and CCL21 in the thymic microenvironment of TAMG patients may lead to autoimmune tolerance defects, serving as core links in the pathogenesis of TAMG (8).

Genetic studies have found that TAMG patients possess a unique HLA allele profile, such as HLA-A*25, HLA-B*40:01, and HLA-DRB1*16, which may confer genetic susceptibility to thymoma and MG (29). The roles of some non-HLA genes, including PTPN22 and CTLA4, in the pathogenesis of TAMG are also gradually being revealed (8, 29). Furthermore, a small proportion of TAMG patients may have concurrent immunodeficiency, manifesting as recurrent infections, hypogammaglobulinemia, and other symptoms. These patients often carry specific HLA-DRB1 and HLA-DQB1 alleles and have a poorer prognosis, requiring attention from clinicians (30).

### 3.3 Genetic characteristics of other MG subtypes

Anti-MuSK antibody-positive MG (MuSK-MG) accounts for 30–50% of AChR antibody-negative MG cases, with clinical manifestations mainly involving facial, shoulder, and bulbar muscles. This subtype responds poorly to cholinesterase inhibitors but responds well to B cell depletion therapies such as rituximab, suggesting that it has a unique pathogenic mechanism (24). Research has found that more than 90% of MuSK-MG patients are mediated by IgG4 subclass antibodies, which is markedly different from conventional MG (mainly IgG1 and IgG3). Further analysis revealed that the total serum IgG4 level was elevated in MuSK-MG patients but had no significant correlation with the MuSK antibody titer, suggesting that IgG4 antibodies may have an antigen-nonspecific mechanism of action in the pathogenesis of MuSK-MG (31). The latest research has also discovered that MuSK-MG is associated with a specific HLA-DR14-DQ5 haplotype, which may lead to the development of MuSK-MG by influencing the activation of autoreactive T cells and IgG4 class switching (4, 32).

### 3.4 Genetic characteristics of the ocular MG subtype

Ocular MG is a localized form of MG characterized by ptosis and diplopia, with a relatively mild natural course. Genetic studies have found that ocular MG is associated with HLA-DQ and PTPN22 loci, but the overall genetic burden is lighter than that of generalized MG (4). Additionally, a small proportion of severe generalized MG patients are resistant to various conventional immunotherapies and are referred to as refractory MG. The latest research has found that the proportion of follicular helper T cells (Tfh) is increased in refractory MG patients, and serum CXCL13 levels are significantly elevated, suggesting that Tfh cell-mediated humoral immune abnormalities may be an important cause of the development and treatment resistance of this subtype (33).

## 4 Exploration of gene–environment interactions and epigenetic mechanisms in MG

### 4.1 Gene–environment interaction patterns in MG

Research on DJ-1 protein provides a new perspective for elucidating the gene–environment interaction mechanisms of MG. DJ-1 is a multifunctional protein that plays an important role in neurodegenerative diseases. Studies have found that DJ-1 gene mutations, abnormal expression, and post-translational modifications can promote the occurrence of various neurodegenerative diseases through mechanisms such as mitochondrial dysfunction, oxidative stress, and autophagy defects (34). Since DJ-1 also plays a key role in neuromuscular junction protection, it is speculated that its abnormalities may mediate the development of MG through similar mechanisms. This suggests that certain MG-related genes may have the dual characteristics of genetic susceptibility and environmental response, serving as a “bridge” for gene–environment interactions.

### 4.2 Involvement of abnormal DNA methylation modifications in MG pathogenesis

DNA methylation is an important epigenetic modification that participates in disease development by influencing gene transcriptional activity. In recent years, multiple studies have found extensive DNA methylation abnormalities in the peripheral blood and thymic tissue of MG patients, suggesting its important role in the pathogenesis of MG. These epigenetic modifications specifically affect neuromuscular junction function through several critical mechanisms:

1. DNA methylation changes in the promoter regions of acetylcholine receptor subunit genes directly influence receptor density at the neuromuscular junction. For instance, hypomethylation of the CHRNA1 promoter leads to increased expression of AChR *α*-subunits (28), directly affecting synaptic transmission efficiency.
2. Histone modifications, particularly H3K27ac and H3K4me3 marks, regulate the accessibility of key genes involved in neuromuscular transmission. Studies have shown that alterations in these modifications affect the expression of proteins crucial for synaptic maintenance and function (28, 35).
3. The interplay between these epigenetic mechanisms and neuromuscular junction proteins creates a regulatory network that maintains synaptic homeostasis. Disruption of this network through aberrant epigenetic modifications can lead to compromised neuromuscular transmission and MG symptoms (36).

Researchers used the Illumina 850 K methylation chip to compare the DNA methylation profiles of 8 MG patients and 4 healthy controls, finding significantly reduced methylation levels of genes such as calcium/calmodulin-dependent protein kinase 1D (CAMK1D) and cAMP response element-binding protein 5 (CREB5) in the MG group. Further experiments showed that hypomethylation of the promoter regions of CAMK1D and CREB5 was associated with their upregulated expression, suggesting that these two genes may play an important role in the pathogenesis of MG through DNA methylation abnormalities (28). This study was the first to reveal the DNA methylation landscape of MG at the whole-genome level, providing important clues for subsequent in-depth research.

The NLRP3 inflammasome is closely related to various autoimmune diseases. Studies have found that polymorphisms in the gene encoding NLRP3 are significantly associated with MG, and carriers of the rs3806265 C allele have a significantly increased risk of developing MG (35). Research indicates that thymoma-associated MG (TAMG) has unique molecular genetic characteristics. However, there were no significant differences in the methylation levels of repair and tumor suppressor genes such as DNA methyltransferase (MGMT) and cyclin-dependent kinase inhibitor 2A (CDKN2A) between TAMG tissues and non-myasthenia gravis thymoma tissues, suggesting that methylation abnormalities of these genes may not be key events in the pathogenesis of TAMG (36).

### 4.3 Regulation of MG development by histone modifications and non-coding RNAs

In addition to DNA methylation, histone modifications and non-coding RNAs are also important epigenetic regulatory mechanisms of gene expression. The complement regulatory protein CD59 can inhibit the formation of the membrane attack complex and plays an important protective role in neuromuscular junction immune damage. Studies have found that CD59 expression is significantly increased in the skeletal muscle tissue of MG patients and is closely related to clinical severity. Further analysis showed that the upregulation of CD59 mRNA and protein levels may be a compensatory protection mechanism against complement-mediated membrane damage (37). This provides a new entry point for the treatment of MG, namely enhancing CD59 expression to inhibit excessive complement activation and reduce neuromuscular junction damage.

Non-coding RNAs are a hotspot in epigenetic research. Circular RNAs (circRNAs) have characteristics such as high stability and tissue-specific expression, showing great application potential in the diagnosis and treatment of nervous system diseases. Studies have found that hsa-circRNA5333-4 is significantly upregulated in the peripheral blood of MG patients and is closely related to MG scale scores, making it a promising new marker for monitoring MG disease progression and treatment efficacy (38). Additionally, the roles of miRNAs such as miRNA-320a, let-7, and miR-150-5p in the pathogenesis of MG have been successively discovered (39, 40). These studies have laid the foundation for elucidating the non-coding RNA regulatory network of MG.

In summary, various epigenetic mechanisms such as DNA methylation, histone modifications, and non-coding RNAs play key roles in the pathogenesis of MG and are closely related to genetic factors and environmental exposures. With the rapid development of epigenomics, RNA-omics, and other disciplines, the understanding of the gene–environment-epigenetic interaction patterns in MG will continue to deepen, providing new breakthroughs for MG etiological research.

## References

[1] Bubuioc A Kudebayeva A Turuspekova S. The epidemiology of myasthenia gravis. J Med Life. (2021) 14:7–16. doi: 10.25122/jml-2020-0145, PMID: 33767779 PMC7982252

[2] Gilhus NE. Myasthenia gravis and congenital myasthenic syndromes. Handb Clin Neurol. (2023) 195:635–52. doi: 10.1016/B978-0-323-98818-6.00010-8, PMID: 37562891

[3] Tannemaat MR Huijbers MG Verschuuren J. Myasthenia gravis-pathophysiology, diagnosis, and treatment. Handb Clin Neurol. (2024) 200:283–305. doi: 10.1016/B978-0-12-823912-4.00026-8, PMID: 38494283

[4] Deymeer F. Myasthenia gravis: MuSK MG, late-onset MG and ocular MG. Acta Myol. (2020) 39:345–52. doi: 10.36185/2532-1900-038, PMID: 33458590 PMC7783433

[5] Punga AR Maddison P Heckmann JM Guptill JT Evoli A. Epidemiology, diagnostics, and biomarkers of autoimmune neuromuscular junction disorders. Lancet Neurol. (2022) 21:176–88. doi: 10.1016/S1474-4422(21)00297-0, PMID: 35065040

[6] Mantegazza R Bernasconi P Cavalcante P. Myasthenia gravis: from autoantibodies to therapy. Curr Opin Neurol. (2018) 31:517–25. doi: 10.1097/WCO.0000000000000596, PMID: 30156572

[7] Yasumizu Y Ohkura N Murata H Kinoshita M Funaki S Nojima S. Myasthenia gravis-specific aberrant neuromuscular gene expression by medullary thymic epithelial cells in thymoma. Nat Commun. (2022) 13:4230. doi: 10.1038/s41467-022-31951-8, PMID: 35869073 PMC9305039

[8] Dalakas MC. Immunotherapy in myasthenia gravis in the era of biologics. Nat Rev Neurol. (2019) 15:113–24. doi: 10.1038/s41582-018-0110-z, PMID: 30573759

[9] Chia R Saez-Atienzar S Murphy N. Identification of genetic risk loci and prioritization of genes and pathways for myasthenia gravis: a genome-wide association study. Proc Natl Acad Sci U S A. (2022) 119:e2206754119. doi: 10.1073/pnas.2206754119, PMID: 35074870 PMC8812681

[10] Liu C Mao C Li S Su Y Liu H Wang X. Myasthenia gravis and ischemic stroke: a bidirectional Mendelian randomization study. Curr Neurovasc Res. (2023) 20:270–9. doi: 10.2174/1567202620666230703122140, PMID: 37403387

[11] Green JD Barohn RJ Bartoccion E Benatar M Blackmore D Chaudhry V. Epidemiological evidence for a hereditary contribution to myasthenia gravis: a retrospective cohort study of patients from North America. BMJ Open. (2020) 10:e37909. doi: 10.1136/bmjopen-2020-037909, PMID: 32948566 PMC7511637

[12] Topaloudi A Zagoriti Z Flint AC Martinez MB Yang Z Tsetsos F. Myasthenia gravis genome-wide association study implicates AGRN as a risk locus. J Med Genet. (2022) 59:801–9. doi: 10.1136/jmedgenet-2021-107953, PMID: 34400559

[13] Li K Ouyang Y Yang H. Myasthenia gravis and five autoimmune diseases: a bidirectional Mendelian randomization study. Neurol Sci. (2024) 45:1699–706. doi: 10.1007/s10072-023-07163-3, PMID: 37910321

[14] Li J Wang F Li Z Feng J Men Y Han J. Integrative multi-omics analysis identifies genetically supported druggable targets and immune cell specificity for myasthenia gravis. J Transl Med. (2024) 22:302. doi: 10.1186/s12967-024-04994-2, PMID: 38521921 PMC10960998

[15] Zhong H Huan X Zhao R Su M Yan C Song J. Peripheral immune landscape for hypercytokinemia in myasthenic crisis utilizing single-cell transcriptomics. J Transl Med. (2023) 21:564. doi: 10.1186/s12967-023-04421-y, PMID: 37620910 PMC10464341

[16] Liu P Qi G Gu S Dong H Liu C Yang H. Single-cell transcriptomics and network pharmacology reveal therapeutic targets of Jianpi Yiqi Bugan Yishen decoction in immune cell subsets of children with myasthenia gravis. Transl Pediatr. (2022) 11:1985–2003. doi: 10.21037/tp-22-593, PMID: 36643680 PMC9834954

[17] Tian DS Qin C Dong MH Heming M Zhou LQ Wang W. B cell lineage reconstitution underlies CAR-T cell therapeutic efficacy in patients with refractory myasthenia gravis. EMBO Mol Med. (2024) 16:966–87. doi: 10.1038/s44321-024-00043-z, PMID: 38409527 PMC11018773

[18] Ouyang Y Chen Y Chen K Tang Z Shi G Qu C. Mendelian randomization and colocalization analysis reveal novel drug targets for myasthenia gravis. Hum Genomics. (2024) 18:43. doi: 10.1186/s40246-024-00607-7, PMID: 38659056 PMC11040902

[19] Meng HY Li X Jin WL Yan CK Dong XH Xu Q. Multiple genetic factors affecting the pharmacokinetic and pharmacodynamic processes of tacrolimus in Chinese myasthenia gravis patients. Eur J Clin Pharmacol. (2020) 76:659–71. doi: 10.1007/s00228-019-02803-0, PMID: 31955224

[20] Bo C Zhang H Cao Y Lu X Zhang C Li S. Construction of a TF-miRNA-gene feed-forward loop network predicts biomarkers and potential drugs for myasthenia gravis. Sci Rep. (2021) 11:2416. doi: 10.1038/s41598-021-81962-6, PMID: 33510225 PMC7843995

[21] Cao Y Lu X Wang J Zhang H Liu Z Xu S. Construction of an miRNA-regulated drug-pathway network reveals drug repurposing candidates for myasthenia gravis. Int J Mol Med. (2017) 39:268–78. doi: 10.3892/ijmm.2017.2853, PMID: 28075449 PMC5358695

[22] Creary LE Gangavarapu S Caillier SJ Cavalcante P Frangiamore R Lie BA. Next-generation sequencing identifies extended HLA class I and II haplotypes associated with early-onset and late-onset myasthenia gravis in Italian, Norwegian, and Swedish populations. Front Immunol. (2021) 12:667336. doi: 10.3389/fimmu.2021.667336, PMID: 34163474 PMC8215161

[23] Salvado M Caro JL Garcia C Rudilla F Zalba-Jadraque L Lopez E. HLA-DQB1*05:02, *05:03, and *03:01 alleles as risk factors for myasthenia gravis in a Spanish cohort. Neurol Sci. (2022) 43:5057–65. doi: 10.1007/s10072-022-06102-y, PMID: 35524016

[24] Borges LS Richman DP. Muscle-specific kinase myasthenia gravis. Front Immunol. (2020) 11:707. doi: 10.3389/fimmu.2020.00707, PMID: 32457737 PMC7225350

[25] Yang WH Lu YR Qiu L Ou CY Lin ZZ Huang ZD. Analysis of clinical characteristics and related genetic variation of juvenile myasthenia gravis. Zhonghua Yi Xue Za Zhi. (2022) 102:1445–9. doi: 10.3760/cma.j.cn112137-20210904-02019, PMID: 35599409

[26] Özsoy Ö Cinleti T Günay Ç Sarıkaya G Giray Ö Çağlayan A. Genetic, serological and clinical evaluation of childhood myasthenia syndromes- single center subgroup analysis experience in Turkey. Acta Neurol Belg. (2023) 123:2325–35. doi: 10.1007/s13760-023-02370-3, PMID: 37656362

[27] Burnet M. Role of the thymus and related organs in immunity. Br Med J. (1962) 2:807–11. doi: 10.1136/bmj.2.5308.807, PMID: 13874953 PMC1926280

[28] Altinonder I Kaya M Yentur SP. Thymic gene expression analysis reveals a potential link between HIF-1A and Th17/Treg imbalance in thymoma associated myasthenia gravis. J Neuroinflammation. (2024) 21:126. doi: 10.1186/s12974-024-03095-7, PMID: 38734662 PMC11088784

[29] Shichkin VP Antica M. Key factors for Thymic function and development. Front Immunol. (2022) 13:926516. doi: 10.3389/fimmu.2022.926516, PMID: 35844535 PMC9280625

[30] Ishizuchi K Takizawa T Ohnuki Y Sekiguchi K Motegi H Oyama M. Immunodeficiency in patients with thymoma-associated myasthenia gravis. J Neuroimmunol. (2022) 371:577950. doi: 10.1016/j.jneuroim.2022.577950, PMID: 35994947

[31] Vergoossen D Ruiter AM Keene KR. Enrichment of serum IgG4 in MuSK myasthenia gravis patients. J Neuroimmunol. (2022) 373:577978. doi: 10.1016/j.jneuroim.2022.577978, PMID: 36240543

[32] Huijbers MG Verschuuren J. Treating muscle-specific kinase myasthenia gravis from the inside out. Neurol Neuroimmunol Neuroinflamm. (2020) 7:e646. doi: 10.1212/NXI.0000000000000646, PMID: 31831572 PMC6935833

[33] Nguyen T Phan CL Supsupin EJ. Therapeutic and diagnostic challenges in myasthenia gravis. Neurol Clin. (2020) 38:577–90. doi: 10.1016/j.ncl.2020.03.005, PMID: 32703470

[34] Huang M Chen S. DJ-1 in neurodegenerative diseases: pathogenesis and clinical application. Prog Neurobiol. (2021) 204:102114. doi: 10.1016/j.pneurobio.2021.102114, PMID: 34174373

[35] Agah E Nafissi S Saleh F Sarraf P Tafakhori A Mousavi SV. Investigating the possible association between NLRP3 gene polymorphisms and myasthenia gravis. Muscle Nerve. (2021) 63:730–6. doi: 10.1002/mus.27193, PMID: 33533549

[36] Coppede F Ricciardi R Lopomo A. Investigation of MLH1, MGMT, CDKN2A, and RASSF1A gene methylation in Thymomas from patients with myasthenia gravis. Front Mol Neurosci. (2020) 13:567676. doi: 10.3389/fnmol.2020.567676, PMID: 33192293 PMC7645111

[37] Iwasa K Furukawa Y Yoshikawa H Yamada M Ono K. CD59 expression in skeletal muscles and its role in myasthenia gravis. Neurol Neuroimmunol Neuroinflamm. (2023) 10:e200057. doi: 10.1212/NXI.0000000000200057, PMID: 36396448 PMC9747141

[38] Lv J Ren L Han S Zhang J Zhao X Zhang Y. Peripheral blood hsa-circRNA5333-4: a novel biomarker for myasthenia gravis. Clin Immunol. (2021) 224:108676. doi: 10.1016/j.clim.2021.108676, PMID: 33465495

[39] Ao W Tian C He X Hu Y Wang W Liu Y. Upregulation of miR150-5p in generalized myasthenia gravis patients is associated with decreased serum levels of IL-17 and increased serum levels of IL-10. Biomed Pap Med Fac Univ Palacky Olomouc Czech Repub. (2020) 164:57–62. doi: 10.5507/bp.2019.009, PMID: 30945700

[40] Wang X Zhang H Lu X Li S Kong X Liu L. LncRNA OIP5-AS1 modulates the proliferation and apoptosis of Jurkat cells by sponging miR-181c-5p to regulate IL-7 expression in myasthenia gravis. PeerJ. (2022) 10:e13454. doi: 10.7717/peerj.13454, PMID: 35602889 PMC9121865

